# Identification of an Isothiocyanate on the HypEF Complex Suggests a Route for Efficient Cyanyl–Group Channeling during [NiFe]–Hydrogenase Cofactor Generation

**DOI:** 10.1371/journal.pone.0133118

**Published:** 2015-07-17

**Authors:** Sven T. Stripp, Ute Lindenstrauss, R. Gary Sawers, Basem Soboh

**Affiliations:** 1 Department of Physics, Freie Universität Berlin, Berlin, Germany; 2 Institute of Microbiology, Martin–Luther University Halle–Wittenberg, Halle (Saale), Germany; Louisiana State University and A & M College, UNITED STATES

## Abstract

[NiFe]–hydrogenases catalyze uptake and evolution of H_2_ in a wide range of microorganisms. The enzyme is characterized by an inorganic nickel/ iron cofactor, the latter of which carries carbon monoxide and cyanide ligands. *In vivo* generation of these ligands requires a number of auxiliary proteins, the so–called Hyp family. Initially, HypF binds and activates the precursor metabolite carbamoyl phosphate. HypF catalyzes removal of phosphate and transfers the carbamate group to HypE. In an ATP–dependent condensation reaction, the *C*–terminal cysteinyl residue of HypE is modified to what has been interpreted as thiocyanate. This group is the direct precursor of the cyanide ligands of the [NiFe]–hydrogenase active site cofactor. We present a FT–IR analysis of HypE and HypF as isolated from *E*. *coli*. We follow the HypF–catalyzed cyanation of HypE *in vitro* and screen for the influence of carbamoyl phosphate and ATP. To elucidate on the differences between HypE and the HypEF complex, spectro–electrochemistry was used to map the vibrational Stark effect of naturally cyanated HypE. The IR signature of HypE could ultimately be assigned to isothiocyanate (–N=C=S) rather than thiocyanate (–S–C≡N). This has important implications for cyanyl–group channeling during [NiFe]–hydrogenase cofactor generation.

## Introduction

Hydrogenases catalyze reversible hydrogen activation in archaea, bacteria, and plants. They are primarily found in the context of anaerobic respiration, fermentation, and photosynthesis [1,2]. The outstanding efficiency of hydrogen evolution with certain hydrogenases explains the interest in this ancient class of metalloproteins; their redox chemistry inspires new ways towards a ‘hydrogen economy’ [3]. The composition of the metal cofactor allows classification into Fe–only, [FeFe]–, and [NiFe]–hydrogenases [4]. The latter class is ubiquitous in prokaryotes, and several crystal structures of these enzymes have been described [5–8]. Their active site cofactor comprises a nickel ion coordinated by four cysteinyl residues. Two of these additionally coordinate an iron ion ligated with two cyanides (CN) and a single carbon monoxide species (CO). [NiFe]–hydrogenases differ in structure, cofactor composition, and enzymatic profile. However the catalytic [NiFe]–(CN)_2_CO site is conserved in all hydrogenases of the [NiFe]–type [9].

Ligation of the iron site with CN and CO requires a complex maturation apparatus, including auxiliary proteins HypC through HypF [10–12]. HypD is the central construction site in cofactor generation and was found to bind a Fe(II)–(CN)_2_CO moiety [13]. In contrast to earlier findings of Lenz and co–workers [14] the results of a recent study suggest that the iron ion on HypD is first modified with CO and then accepts two equivalents of CN from a cyanated cysteine residue at the *C*–terminus of HypE [15,16]. Once formed on the HypD scaffold, the Fe(II)–(CN)_2_CO site is transferred to the hydrogenase apo–protein by a mechanism that is not fully understood but has been shown to require functional HypC [17,18]. HypC forms a very stable complex with HypD, HypCD [14,19–22].

**Fig 1:**
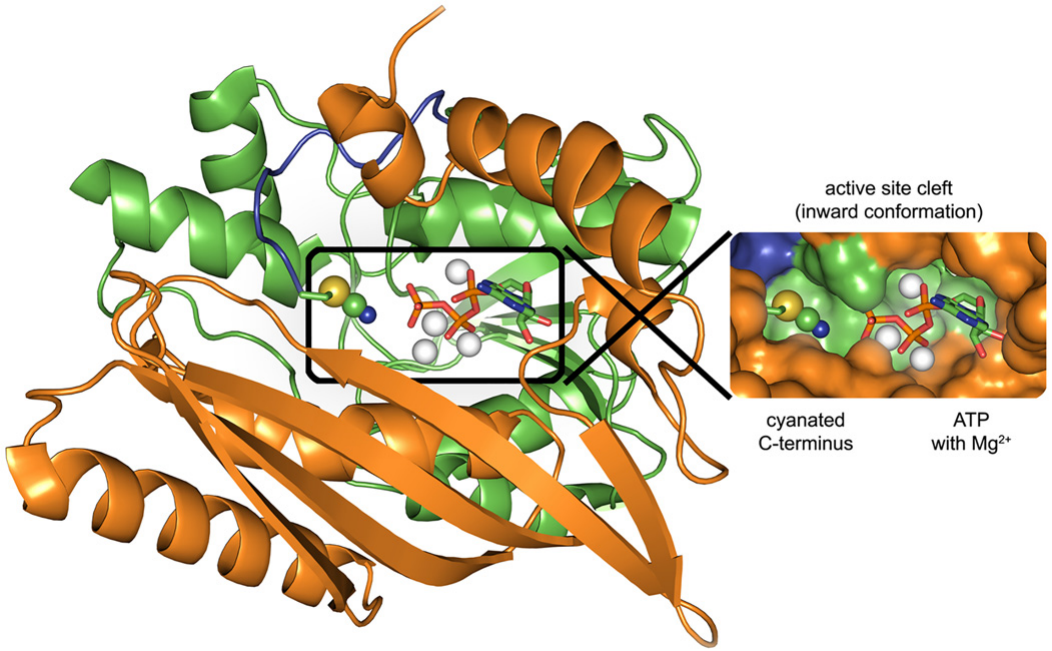
Crystal structure of HypE–SCN from *T*. *kodakarensis* in the ‘inward’ conformation. The αβ domains A and B are colored orange and green, respectively. The *C*–terminal loop region (330–338) is shown in blue. The loop ends with the conserved PR(V/I)C motif and the cysteine (C338) is modified to a thiocyanate in the published model [27]. Inset: domains A and B form a surface cleft that localizes the C–terminus close to a magnesium–sequestered ATP molecule. Drawn after pdb coordinates 3WQJ.

The aforementioned HypE forms a hetero–oligomer with HypCD [19] and a heterodimer with HypF (see below). HypE has been crystallized as a homodimer from different microorganisms [23–25] including *E*. *coli* [26]. Fig 1 shows the 36 kDa monomer with domains A and B forming the active site cleft that binds ATP in proximity to a conserved peptide motif, PR(I/V)C. The *C*–terminal cysteine residue of HypE (C336 in *E*. *coli* nomenclature) is proposed to be modified to thiocyanate [15,22]; however, none of the HypE crystal structures did reveal the cyanated cysteinyl. In 2013, Tominaga and co–workers crystallized carbamoylated and cyanated forms of HypE from *Thermococcus kodakarensis* after *in vitro* incubation of the protein with potassium cyanide and ATP [27]. ATP binding is facilitated by a number of conserved motifs; chief among these is DX_4_GAXP, which defines HypE as a member of the PurM superfamily [28]. In most crystal structures, the presence of ATP correlates with the so–called ‘inward conformation’ in which the *C*–terminal loop is in the proximity of the active site cleft and of the terminal phosphate of ATP [23–27,29]. In the ‘outward conformation’ the *C*–terminus is no longer near the active site cleft but rather protrudes from the protein surface. While the ‘inward conformation’ clearly represents the catalytically active structure, the ‘outward conformation’ has been interpreted to facilitate contact with either HypD or HypF [25,29].

ATP mainly affects the tertiary structure of HypE. In contrast, auxiliary protein HypF has been shown to hydrolyze ATP during catalysis. HypF uses ATP to activate the cyanide precursor carbamoyl phosphate via a carbamoyl adenylate intermediate [15]. The 82 kDa transferase HypF comprises four domains, two of which bind ATP [30]. The structure of the HypEF heterodimer suggests a conformation with the catalytic cysteine of HypE pointed towards AMP (naturally carbamoyl adenylate) at the nucleotide–binding ‘Kae1–like’ domain of HypF [25,30,31]. The *C*–terminal cysteine residue of HypE is carbamoylated at the expense of ATP, and AMP leaves the complex. The ATP–dependent condensation to thiocyanate has been demonstrated *in vitro* [27], however, transfer of HypE–SCN to HypD–Fe has not yet been shown unequivocally. A crystal structure of the HypCDE super–complex has been resolved but electron density at the *C*–terminus of HypE (R336 –C338) was found to be discontinuous [19].

In the current study, we have analyzed HypE and HypF of *E*. *coli* by means of *attenuated total reflection* (ATR) FT–IR spectroscopy. Distinct proof for the natural thiocyanate ligand of HypE is presented. Making use of a novel *in vitro* maturation approach, cyanation of HypE from carbamoyl phosphate and isolated HypF is demonstrated. The observed red–shift of the CN stretching frequency from 2118 cm^–1^ (HypE) to 2105 cm^–1^ (HypEF) can be explained by Stark splitting due to changes in the specific charge distribution of the ligand. This interpretation is confirmed by *surface–enhanced IR absorption spectroscopy* (SEIRAS) and *protein film voltammetry* (PFV) on HypE at an external potential of ±300 mV vs. standard hydrogen (SHE). Interestingly, the FT–IR analysis hints at isothiocyanate (–N = C = S) rather than the previously proposed thiocyanate intermediate (–S–C≡N). The spectro–electrochemical characterization of HypE and HypEF presented here allows us to discuss conformational changes upon complex formation, the influence of the protein fold coordination sphere on the ligand, and the role of ATP in (iso–) thiocyanate formation. Finally, S→N isomerization of thiocyanate is discussed in the context of protein/ protein interaction and substrate channeling.

## Materials and Methods

His–tagged HypD, HypE, and HypF proteins were purified as described previously [21] except that 10 mM magnesium acetate was included in all buffers used for purification. The functional holoenzyme of the regulatory hydrogenases of *Ralstonia eutropha* (*Re*RH) was heterogeneously synthesized and isolated via Strep–tag as documented by Buhrke and co–workers [32]. We use the regulatory hydrogenase of *R*. *eutropha* as a well–characterized example of the active [NiFe]–(CN)_2_CO cofactor [33].

For ATR FT–IR analysis 1 μL HypD and HypE (each 10 g/L) was concentrated individually on top of a silicon crystal with two active reflections in an ATR set–up (Smith Detection, Warrington, USA) using a fast–scan Tensor27 spectrometer (Bruker Optik, Ettlingen, Germany). The solution was deprived of water by a constant stream of dry N_2_ gas. The IR spectrum of *Re*RH was recorded on a humidified film of 1 μL of 20 g/L to prevent active site degradation. Water concentration in the film is controlled by the volume of N_2_ gas running through a fixed contingent of liquid water as described earlier [20]. Relative humidity was adjusted to >95% (Voltcraft, Wollerau, Switzerland) for 1 atm and room temperature (24°C). The Ni–S state of the as–isolated sample [33] was enriched by continuous degassing with a humidified N_2_. All samples were handled under a protective N_2_ atmosphere and in the absence of O_2_. All spectra were recorded with a spectral resolution of 4 cm^–1^ and varying number of interferogram scans per spectrum.

We analyzed HypE by means of SEIRAS. A single–reflection silicon prism in an ATR set–up was modified with a gold thin film of nano–scale roughness as described earlier [34]. In the vicinity of the surface the gold island structure gives rise to plasmon enhancement thus monolayers of protein can be analyzed without contribution from the bulk solution. SEIRAS was performed in the absence of O_2_ under a N_2_ atmosphere with 1% H_2_. 3 μg HypE in 200 μL 10 mM KP_i_ pH 7 were incubated directly with the gold surface for 6–18 h. Protein film formation was followed by FT–IR spectroscopy. When the amide II band (1549 cm^–1^) did not increase any further (typically up to 3 x 10^-3^ absorbance units, ΔA) the buffer was exchanged to electrolyte solution (10 mM KP_i_ pH 7 with an additional concentration of 150 mM NaCl and 20 mM MgCl_2_). The cell was equipped with a platinum counter electrode and Ag/ AgCl reference electrode. The gold film is exploited as working electrode by a joined PARSTAT 2273 potentiostat (Princeton Applied Research, Oak Ridge, USA). Potential–induced difference spectra at –300 and +300 mV vs. SHE were recorded on a background of HypE (10.000 interferogram scans per spectrum with a resolution of 4 cm^–1^).

To elucidate the effects of HypF, carbamoyl phosphate, sodium dithionite, and ATP on HypE a simple *in vitro* assay was established. Both carbamoyl phosphate and ATP stock solutions were prepared freshly for every experiment. ATP was dissolved in 10 mM sodium phosphate buffer (pH 6 ± 0.1) and in the presence of 20 mM MgCl_2_. All experiments were conducted at 4°C in 10 mM sodium phosphate buffer pH 7 ± 0.1 with 10 mM MgCl_2_, 500 μM sodium dithionite, and under oxygen–free conditions. Phosphatase activity of HypF and complex formation with HypE were verified by native PAGE as described earlier [35,36]. In the reaction mixture a HypE/ HypF ratio of 1:1 rendered good results. The highest tested concentration of carbamoyl phosphate was found to be 100 μM (higher concentration caused visible precipitation). The influence of ATP was analyzed up to a concentration of 1 mM. With all components mixed in a pre–cooled 200 μL PCR tube the reaction was started by adding HypF. After various time points of incubation (5, 10, 20, 30, 60, 120, and 180 minutes) 1 μL of the solution was transferred to the pre–cooled ATR cell, rapidly dried under N_2_, and probed against a background of HypE (10.000 scans with 4 cm^–1^ spectral resolution).

## Results

**Fig 2:**
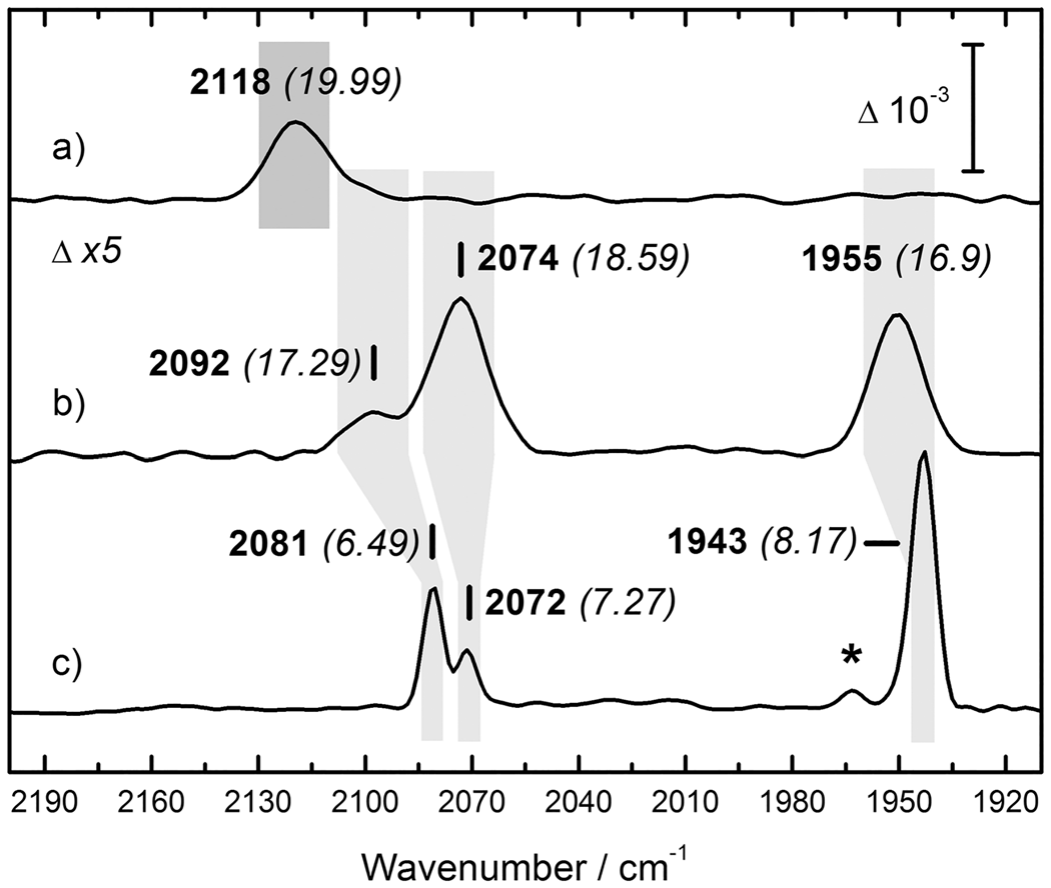
ATR FT–IR analysis of HypE, HypD, and ReRH. Spectrum a) shows the rhodanide signature of HypE from *E*. *coli* at 2118 cm^–1^. Isolated from the same organism, spectrum b) shows the Fe(II)–(CN)_2_CO absorbance of HypD as discussed earlier [13]. Spectrum c) shows the Ni–S active site signature of the regulatory hydrogenase from R. eutropha, ReRH [33]. The asterisk at 1961 cm^–1^ indicates a minor fraction of Ni–C. Midpoint frequencies are given in bold wavenumbers, FWHM of the fitted Gaussian is in cursive brackets. The width of the grey boxes represents peak width and is plotted for illustration. All samples were probed with a spectral resolution of 4 cm^–1^.

Fig 2 shows the ATR FT–IR spectrum of HypE in the spectral range between 2200 and 1900 cm^–1^ (a). Signals are normalized to amide II band height and corrected by a spline function that reproduces the broad, underlying ‘combination’ band of liquid water [37]. HypE displays a small yet defined peak at a midpoint frequency of 2118 cm^–1^. With a *full width at half maximum* (FWHM) of 20 cm^–1^ the peak is rather broad. Moreover, Fig 2 includes the Fe(II)–(CN)_2_CO signature of HypD and *Re*RH. The peaks at 2092/ 2074 and 2081/ 2072 cm^–1^ have been assigned to Fe(II)–(CN)_2_ while band contributions at 1943 and 1955 cm^–1^ stem from the asymmetric stretch vibration of Fe(II)–CO [13,33]. A small fraction of Ni–C in the *Re*RH spectrum (spectrum c) is suggested by the asterisk at 1961 cm^–1^ [33]. Notably, the IR spectrum of the functional *Re*RH active site is sharper by a factor of two to three, based on the FWHM of Gaussian fits for Fe–CN and Fe–CO (breadth given in brackets in Fig 2). This has been discussed to be due to a higher degree of conformational freedom of CO and CN on HypD and a tighter hydrogen–bonding network in active hydrogenases [20].

**Fig 3:**
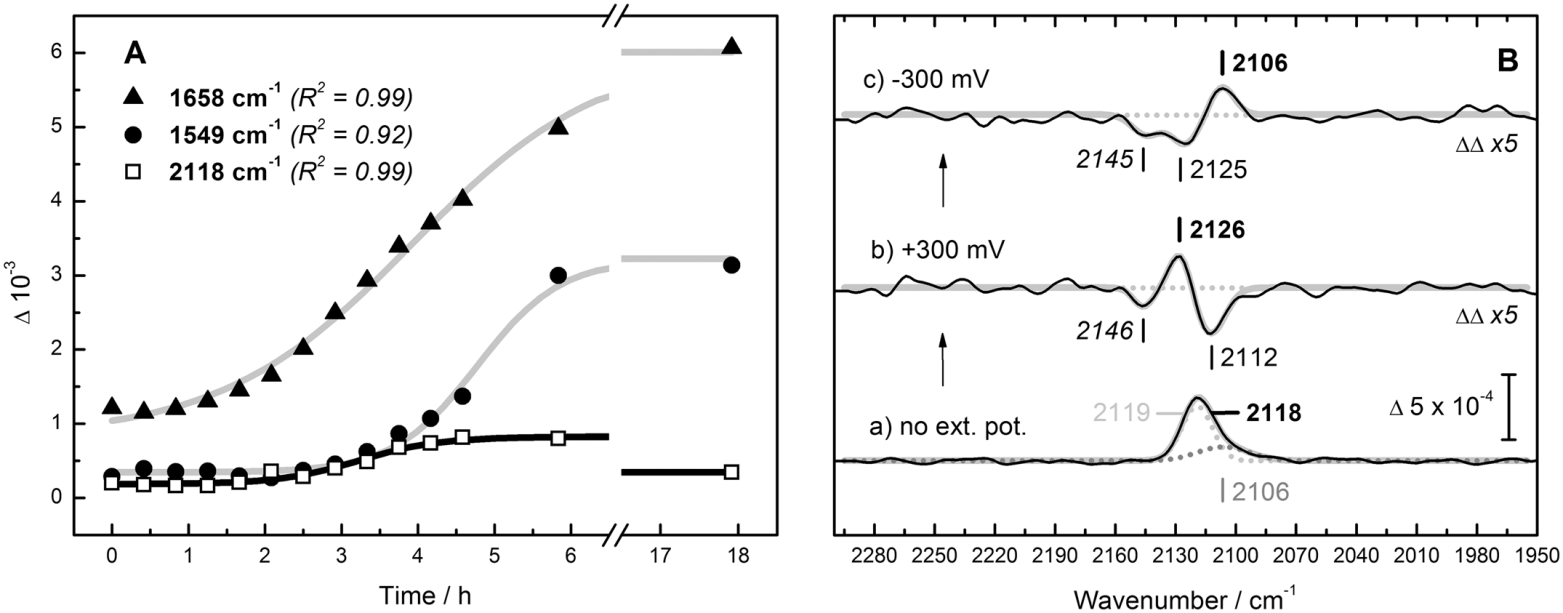
SEIRAS probes the vibrational Stark effect of HypE. **(A)** Amide I (▲, 1658 cm^–1^) and amide II (●, 1549 cm^–1^) band formation over time after injection of HypE onto the bare gold surface. Additionally, the increase of the peak at 2118 cm^–1^ (**□**) is followed. Kinetics are consistent with the Boltzmann model for a sigmoidal fit (R^2^ as given in cursive brackets does not include the 18 h signal for 2118 cm^–1^). **(B)** SEIRAS spectrum of HypE from 2250 to 2000 cm^–1^ without external potential including the thiocyanate vibration at 2118 cm^–1^ (a). The peak fits best with contributions at 2119 and 2106 cm^–1^. Sequentially setting the cell potential to (b) +300 mV and (c) –300 mV vs. SHE gives rise to difference bands illustrating the vibrational Stark effect on HypE. Positive contributions are marked in bold. See text for details. Spectra (b) and (c) fit with three Gaussians to R = 8 x 10^-6^ and 9 x 10^-6^, respectively.

Fig 3 shows the results of the SEIRAS analysis. Fig 3A tracks the increase of bands at 1658 and 1549 cm^–1^ (amide I and II, respectively) over time. This corresponds to formation of a protein monolayer at the gold surface, a typically slow process [34]. After six hours the protein signal is basically stable and amide II was found to be maximal (band height of around ΔA = 3 x 10^-3^). Over the course of time, a single peak appears around 2118 cm^–1^. The band increases simultaneously with the protein signal, however, after five to six hours the signal starts to weaken. Thus, experiments were performed after six hours of protein binding. Fig 3B shows the SEIRA spectrum of HypE in the spectral range between 2250 and 2000 cm^–1^ recorded without external potential (a). Similar to the peak observed in the ATR FT–IR spectrum of HypE in Fig 2a), the band is broad (FWHM = 21.5 cm^–1^). It fits best with two Gaussians centered at 2119 cm^–1^ and 2106 cm^–1^. The later contributes ca. 2% to the integrated area. SEIRAS is a spectro–electrochemical technique that allows an investigation of the influence of an electrical field on a given sample. The thiocyanate ligand as proposed for mature HypE [15] would react to the field with a qualitative change in absorption, the so–called Stark shift [38]. Double difference spectra illustrate the effect of external potentials as HypE was sequentially subjected to +300 mV and –300 mV vs. SHE. Amperometry and cyclic voltammetry did not indicate any redox current. Fig 3B illustrates a vibrational Stark effect on the 2118 cm^–1^ band including a shift to 2126 cm^–1^ at +300 mV (b) and 2106 cm^–1^ for –300 mV (c). Negative contributions of the derivative–shaped signal fit to 2112 cm^–1^ (+300 mV) and 2125 cm^–1^ for –300 mV. The potential–induced difference spectra comprise an additional feature around 2145 cm^–1^, which is negative irrespective of the applied potential.

**Fig 4:**
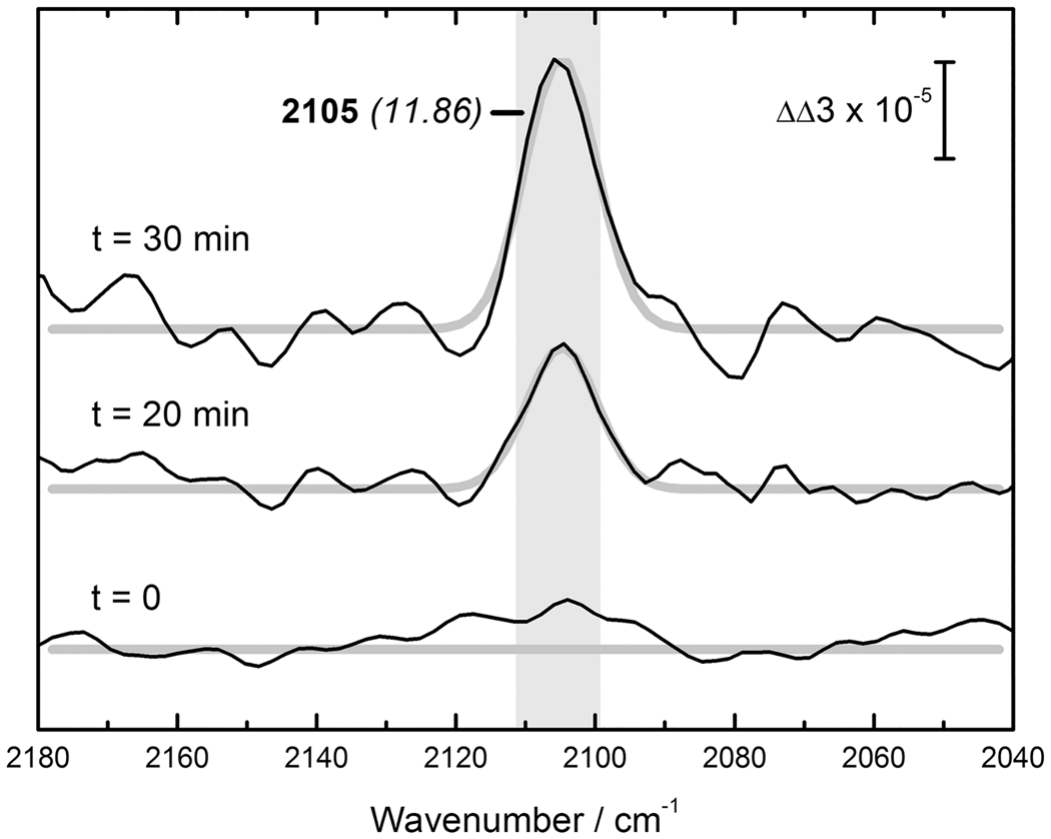
ATR FT–IR analysis of the modification of HypE by HypF and carbamoyl phosphate. The HypEF reaction mix was probed against a background of HypE alone. After t = 0, 20, and 30 minutes, a sharp peak appears at 2105 cm^–1^ (FWHM ≈ 12 cm^–1^, grey box). The signal was found to decrease after 30 minutes. Incubation without carbamoyl phosphate (CP) did not result in peak formation (dashed line).

Fig 4 shows ATR FT–IR double difference spectra of HypEF plus carbamoyl phosphate minus HypE for t = 0, 20, and 30 minutes. By subtraction of HypE, the population naturally carrying the thiocyanate signature (compare Figs 2 and 3B) is masked. A new peak formed that fits to a sharp Gaussian of FWHM ≈ 12 cm^–1^ and a midpoint frequency of 2105 cm^–1^. From 20 to 30 minutes the peak area roughly doubled. No further increase of the peak integral was detected after 60, 120, or 180 minutes. Unspecific changes in the amide region for t = +60 minutes suggest structural inhomogeneities possibly related to a decrease of catalytic competence, if not sample degradation. Both carbamoyl phosphate and HypF were essential for the observed *in vitro* modification of HypE, as no peak formed at lower concentrations, and longer incubations times could not compensate for the lack of substrate. Fig 4 includes the difference spectrum after 30 minutes without carbamoyl phosphate in the reaction mixture (dashed line). Addition of ATP had no positive effect on the cyanation reaction. Moreover, a concentration of ATP higher than 100 μM caused visible precipitation in the reaction mixture, especially if HypF was probed individually. No peak formation was observed in assays including degraded HypE, or HypF.

## Discussion

We present a FT–IR spectroscopic identification of the naturally cyanated cysteinyl residue of HypE isolated from *E*. *coli*. Using two independent FT–IR techniques we detected a broad peak centered at 2118 cm^–1^. In ATR FT–IR, the probing beam is injected from the bottom into an infrared–transparent crystal such as germanium, diamond, zinc selenide or silicon. The beam is reflected towards the detection unit and produces an evanescent wave at the crystal surface that interacts with deposited media [39]. The penetration depth critically varies with the composition of the reflecting crystal. Surface–enhanced IR absorption spectroscopy (SEIRAS) makes use of plasmon resonance signal amplification at a nano–structured gold surface [40]. The gold layer is deposited on top of a silicon or germanium crystal, and the probing beam is applied in ATR configuration. Besides its superior spectral sensitivity, SEIRAS allows for spectro–electrochemistry [34]. Here, the gold layer can be exploited as working electrode and the effect of field gradients or redox titrations can be monitored by voltammetry and FT–IR simultaneously.

### Vibrational assignment

The observed band in the ATR FT–IR and SEIRA spectra of HypE disagrees by at least 20 cm^–1^ with the C–N stretching frequency as expected for a thiocyanate–modified terminus [41,42]. Organic thiocyanates are not found to absorb below 2140 cm^–1^ [43]; however, as thiocyanates are strongly affected by solvation dynamics [44,45] the experimentally observed red–shift on HypE might reflect the influence of the surrounding protein fold. Alternatively, the 2118 cm^–1^ band can be assigned to isothiocyanate. Isothiocyanate is a structural isomer of thiocyanate, and both give rise to slightly different vibrational profiles if bound to Cβ of C336 and the protein backbone. Strong interaction between the cumulative double bonds and the enhanced C–N distance of ~0.3 Å results in a red shift of up to 85 cm^–1^ [41]. In Table 1, stretching frequencies for different cyanate, cyanide, and carbonyl species are compiled.

**Table 1:** (A) Tabulation of typical C–N stretching frequencies of thiocyanates, isothiocyanates, and isocyanates [42,43,46,47]. (B) Iron cyanide and carbonyl stretching frequencies as found with mature [NiFe]–and [FeFe]–hydrogenases [4].

(A)	SCN	NCS	NCO	(B)	CN	CO
CH_3_	2141	2092	2288	Fe(I)	2030–2020	1915–1880
C_2_H_5_	2141	2092	2280	Fe(II)	2090–2060	2010–1930
C_4_H_9_	2137	2088	2280	μFe(II)*	*not observed*	1810–1790

* In oxidized [FeFe]–hydrogenases, one CO ligand can be found in a ‘bridging’ position [48]

To learn more about the absorbent we probed HypE by spectro–electrochemistry in SEIRAS configuration. The breath of the original peak at 2118 cm^–1^ reflects a high order of vibrational freedom; this has been observed for other auxiliary proteins, too [13,49–51]. Fitting suggests two Gaussians with a midpoint frequency at 2119 cm^–1^ and a shoulder at 2106 cm^–1^. High signal intensity results from the uniform orientation of the HypE monolayer and a spatial arrangement of the (iso–) thiocyanate vibration perpendicular to the gold thin film, which enhances absorption due the selection rules that apply in SEIRAS [40]. A direct interaction with the gold surface can be neglected as corresponding shifts were not detected in the spectrum [52]. At ±300 mV vs. SHE a split signal with contribution at 2126 and 2106 cm^–1^ was recorded. The potential as applied across the protein layer causes band shifts that are due to an interaction between the intramolecular charge distribution of the ligand and an external electric field. Infrared absorption critically depends on changes of the dipole moment. Thus, the observed band–splitting is referred to as the vibrational Stark effect [53–55]. While the electrical field applied here is an external one, Stark splitting in proteins is often interpreted to be due to structural rearrangement in the vicinity of the absorbing ligand, e.g. hydrogen–bonding [53]. The SEIRAS experiment proves that the absorbing molecule reacts to an external field and supports the cyanate assignment. Both thiocyanate and isothiocyanate are known to exhibit pronounced Stark splitting [56].

### *In*–*vitro* cyanation of HypE

To follow the modification of HypE *in vitro* we developed an ATR FT–IR assay that includes isolated fractions of HypE and HypF plus carbamoyl phosphate. The absorbent were found to suffer from varying levels of instability that especially complicated the IR analysis of HypE. One explanation for this can be the susceptibility of cyanates for hydrolysis in aqueous media [57]. In the given time frame, however, formation of a peak at 2105 cm^–1^ was shown to be linked with the presence of HypF and carbamoyl phosphate. The preceding experiments allow us to assign this peak to the C–N stretching vibration associated with HypE. Interestingly, the peak is sharp (FWHM = 12.1 cm^–1^) and coincides qualitatively with the signal enriched at –300 mV in the SEIRAS experiment (2106 cm^–1^). Without an external electrical field, this red–shift must arise from changes in the protein environment, thus resulting in band–sharpening and peak shift [38,53,58].

We can assume that the HypEF complex is formed in the reaction mixture [15,22]. Complex formation has been proven to involve the *C*–terminus in particular [25]. The HypEF crystal structure shows that in order to catalyze carbamoylation, HypF forces the *C*–terminal cysteine residue of HypE into a well–defined orientation towards its carbamoyl adenylate substrate. Although our spectroscopic analysis cannot report on complex formation directly, differences between the ‘marker signatures’ at the *C*–terminus of HypE and in the HypEF complex are conceivable. It is informative to consider related protein systems. For example, the vibrational variation between HypD and *Re*RH (Fig 2) stems from a similar coordination sphere difference. In both cases, absorption of a Fe(II)–(CN)_2_CO site is detected, however, for *Re*RH the protein fold facilitates a tighter set of electrostatic interactions. This combination of band–sharpening and qualitative shifts has recently been seen for the insertion of an artificial cofactor into the *Chlamydomonas reinhardtii* [FeFe]–hydrogenase apoprotein [59]. The di–iron cofactor that has been found on the central construction site of the [FeFe]–hydrogenase maturation protein, HydF [49,60], undergoes similar changes upon transfer to the hydrogenase apoprotein [50,51]. HydF is the functional analogue of HypD [14,20].

Interestingly, ATP had no positive effect on the HypEF reaction described in this study. This contrasts the findings of Soboh and co–workers in which purified Hyp protein components were exploited to generate active hydrogenase in crude extracts [21], and the proposals developed earlier [12,15,19]. We assume that both HypE and HypF were isolated in an ATP–bound form [29] that makes additional ATP in the reaction mixture redundant or indeed inhibitory. Experimentally the presence of ATP can be proven in an indirect way. HypE adopts the outward conformation in which the *C*–terminal loop is not positioned in the active site cleft if ATP is absent from the solution [23,24]. Due to the fact that the SEIRAS analysis does not give any indication for the modified *C*–terminus site to react with the gold surface [52], this supports the proposal of a well–protected loop and, accordingly, the presence of ATP on HypE.

## Conclusions

ATR FT–IR analysis of HypE gave rise to a broad band at 2118 cm^–1^, SEIRAS and PFV allowed us to distinguish at least two subspecies, 2125 cm^–1^ and 2105 cm^–1^. The HypEF complex exhibits a sharp peak at 2105 cm^–1^. While the original signal might stem from either thiocyanate or isothiocyanate, the shift to 2105 cm^–1^ in HypEF suggests absorption from the out–of–phase stretching of–N = C = S [41–43] rather than–S–C≡N. The IR spectrum of thiocyanate is dominated by the C–N stretching vibration at higher wavenumbers. Lieber and co–workers analyzed different organic thiocyanates and isothiocyanates and concluded that, “thiocyanate and isothiocyanate can be distinguished by their characteristic vibration frequency around 2140 cm^–1^ and between 2105 and 2060 cm^–1^, respectively” [43]. With thiocyanate and isothiocyanate being ‘linkage isomers’ [61] it is likely that concomitant thiocyanate absorption is detected. Indeed, SEIRA difference spectra show negative peaks around 2145 cm^–1^ that can be tentatively assigned to thiocyanate [41–43].

Different mechanisms drive the S→N isomerization of thiocyanates [62]. For example, thiocyanate is known to trap protons and collapse the pH–gradient across the apical membrane of parietal cells [63]; uncharged isothiocyanic acid (HNCS) permeates biological membrane much faster than the thiocyanate ion [64]. Boxall and Simons pioneered the understanding of HNCS photo–dissociation [65] into H^+^ and thiocyanate under the influence of UV light [66]. Another prominent example of SCN/ NCS isomerization has been described by Buckingham and co–workers as early as 1970 [67]. Here, cobalt–bound thiocyanate undergoes an intramolecular substitution process in which “the N–end acts as nucleophile and the S–end as the leaving group” [68]. This rearrangement proceeds without energetic minima (Palmer and co–workers calculated an activation energy ΔV^╪^ of –5.3 ± 0.8 cm^3^ mol^–1^) and involves a T–shaped transition state suggested both experimentally and by DFT simulations [69–71].

We assume that, in case of HypEF, the thiocyanate–modified *C*–terminus converts into an isothiocyanate by a mechanism as proposed by Buckingham [67] and Rotzinger [68]. Isomerization is driven either via acid/ base chemistry or a sterically more favorable orientation of isothiocyanate at the HypEF interface. While thiocyanate is kinked around the sulfur atom, the C–N = C angle of isothiocyanate is nearly 180° [72] and thus exhibits a strong structural discriminator differentiator reminiscent of the *cis/ trans* isomerization in retinal proteins [73]. Our spectroscopic investigation of HypEF conflicts with the crystal structure of an artificially cyanated HypE homodimer [27] which was modelled under restraints for the kinked thiocyanate isomer. However *in vivo* this very difference might by exploited to avoid aggregation of auxiliary proteins. Both HypF and HypD share HypE as interaction partner thus temporal control is necessary. First, HypF uses carbamoylphosphate to cyanate HypE [27,31]; in complex with HypCD, this cyanate is transferred to the catalytic iron ion of the [NiFe] active site [12,19]. The isothiocyanate–bound HypE as found in complex with HypF can decrease affinity to HypD, thus efficiently inhibiting aggregation of HypCD with *unready* HypE and enabling seamless substrate channeling [74].
